# Nurturing Italian Geo-palaeontological Heritage with Virtual Palaeontology: Preliminary Report of Its Application in Two Natural History Museums

**DOI:** 10.1007/s12371-023-00808-x

**Published:** 2023-03-06

**Authors:** Saverio Bartolini-Lucenti, Lorenzo Rook

**Affiliations:** 1 Earth Science Department, Paleo[Fab]Lab, Università Di Firenze, Via G. La Pira 4, 50121 Florence, Italy; 2 Institut Català de Paleontologia M. Crusafont, Universitat Autònoma de Barcelona, 08193 Cerdanyola del Vallès, Spain; 3 NBFC, National Biodiversity Future Center, Piazza Marina 61 (C/O Palazzo Steri), 90133 Palermo, Italy

**Keywords:** Virtual paleontology, 3D techniques, AR, Digitisation

## Abstract

In this manuscript, we present a preliminary report on the use of virtual palaeontology methodologies in two natural history museums in central Italy, the Geology and Paleontology Museum of Florence and the Civic Museum of Natural Sciences of Faenza. Despite the differences between the museums (in terms of history, type and size of the collections, etc.), the use of surface and computed tomography (CT) scans has allowed the acquisition of a consistent amount of data to create digital copies of selected specimens and to plan several future projects sparked from the use of these methodologies. Our first step focused on the selection of the scanning sample: type and historically relevant specimen in the case of the Florence museum, and fragile and relevant specimens for the Faenza museum from a local yet internationally known site, Cava Monticino, dated to the Late Miocene. The scanning techniques included the use of three high-resolution scanners, with different specifications, to acquire surface data and a medical scanner to obtain CT scans. The outputs of the surface scans were excellent digital copies of the specimens, whereas tomography allowed the first investigations and visualisations of the presence of embedded bones in the fossiliferous blocks of Cava Monticino. The resulting 3D and raw data represent invaluable resources that the two museums are currently planning to implement in their exhibitions using digital visualisation devices and techniques (e.g. AR web apps, touchscreens) or 3D-printed touchable specimens.

## Introduction

The development of virtual palaeontology and its techniques has added numerous arrows to the quiver of natural history museum curators to help them engage people more with their collections and the science behind them. The innovations in the methods for obtaining digital data of fossil specimens now provide easier and less expensive ways for museums and other institutions to use their fossils in exhibits and for conservation purposes. In a way, virtual palaeontology itself has revolutionised how palaeontology is perceived: the dusty, old-fashioned discipline has become a fascinating, rigorous and engaging subject, open to ever-improving techniques and analyses. One key to the success of these methodologies, among many others, lies in the non-invasiveness of their use on the specimens: neither tomographic imaging nor surface scans require preliminary preparation of the specimens, nor do they impose any damage, making them perfect for precious, invaluable objects such as fossils.

As a consequence, natural history museums have derived great benefits by digitising their own collections. For instance, digitising provides the possibility of preserving a virtual copy of a specimen to keep track of its state of conservation. Furthermore, digital archives of stored 3D models of specimens also enable museums to plan restorations of particularly fragile items that require limited handling or special care due either to their value and importance or their intrinsic fragility. Some of these restorations could even be made in the virtual environment, as crushed, deformed or incomplete fossils can be reconstructed, thereby allowing museums to overcome some of the limits imposed by taphonomic and fossilisation processes while offering to the public life-like restorations of long-extinct animals. Recently, mathematical protocols have been applied for analytical retrodeformation of the distorted type specimen of a fossil equid from the Geology and Paleontology Museum of Florence, using geometric morphometric approaches and an undeformed specimen as a guide for the retrodeformation process called *target* (Cirilli et al. 2020). More than 150 years after its first description, the ‘mathematically correct’ original morphology and proportions of this valuable specimen can now be displayed for visitors, as well as used by scholars who intend to study it.

In this ever-developing environment, Italian natural history museums are starting to adopt digital tools for conservation purposes and as implementations for in situ applications or online web pages and apps. In the past few years, the Earth Science Department of the University of Florence has built up a virtual palaeontology lab (formally known as *Paleo[Fab]Lab*) with high-resolution surface scanners (Artec Eva, Artec Spider and Artec Micro) and an SLA 3D printer (Formlab 3). This equipment has been used in different instances for both scientific research and museum conservation purposes. Here, we report two cases of digital applications to the collection of two Italian natural history museums that differ greatly from one another in many aspects (e.g. type of institution, size of their collection, scopes). The first case is the digitisation of the type collection of vertebrates of the Geology and Paleontology Museum of Florence. Thanks to the financial support of the Tuscany Region, a 2-year project named ‘*Paleontologia virtuale, un approccio non invasivo e per la fruizione, diffusione e condivisione del patrimonio paleontologico*’ (acronym PalVirt) was activated, representing the first example in Italy of valorisation of the palaeontological heritage by systematic digitalisation of relevant parts of museum specimens. The second case is the non-invasive investigation of an ossiferous block from the Miocene site of the Monticino gypsum quarry held at the Natural Science Museum of Faenza and its dissemination to the public.

### Geology and Paleontology Museum of Florence

**Fig. 1:**
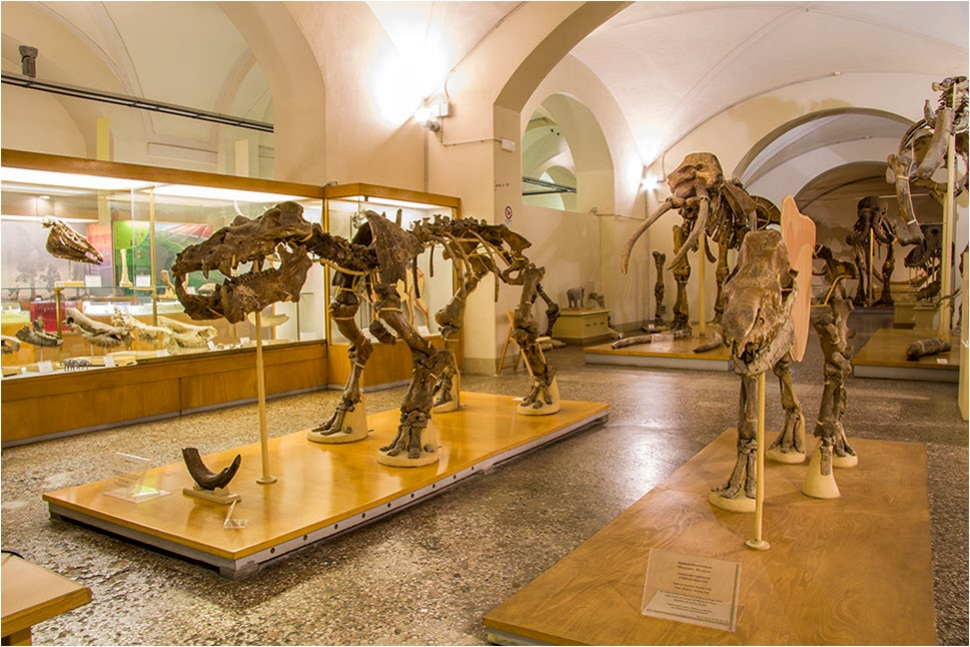
Geology and Paleontology Museum of Florence: here, the renowned Early Pleistocene Upper Valdarno collection, which includes several type specimens

Founded in 1775 as part of the Imperial and Royal Museum of Physics and Natural History, under the illuminated Grand Duke Peter Leopold, the scientific history of this museum spans nearly 250 years. Its high relevance is testified by the presence in its collections of a bulk of fossil specimens collected and known since Medici’s times (i.e. at least since the seventeenth century), as well as by the attention it receives from the international scientific community. Indeed, thanks to the work of important local scientists (e.g. G. Targioni Tozzetti, 1712–1783) in the early nineteenth century, the museum was visited by prestigious scholars like Georges Cuvier (1769–1832), whose observations on the fossils of Upper Valdarno basin were included in its ‘*Recherches sur les ossemens fossiles de quadrupèdes: où l'on rétablit les caractères de plusieurs espèces d'animaux que les révolutions du globe paroissent avoir détruites*’ (1812 and later editions: 1821–1824), and several of its specimens still serve today as international references for extinct taxa (as holotypes, syntypes, etc.) (Monechi and Rook 2010). The museum’s value was also reinforced by the constitution of the Central Palaeontological Collection of Italy in 1861 as a place to store all relevant fossil occurrences of the Peninsula. Unfortunately, the project was abandoned when the Royal Geological Office (now the Geological Survey of Italy) of the Italian Geological Society was established in 1873, but the extensive invertebrate and palaeobotanical collections are still housed in the museum building. Currently, the museum (Fig. 1) holds more than two hundred thousand palaeontological specimens, spanning from the early Palaeozoic to the late Quaternary. The museum was the object of a 2-year project for digitisation and valorisation of its most valuable specimens (e.g. types; historical and iconic specimens), carried out and supervised by the authors of this contribution. This project represents the first attempt at systematic digitisation of palaeontological collections in Italy.

### Museo Civico di Scienze Naturali ‘Malmerendi’ of Faenza

**Fig. 2:**
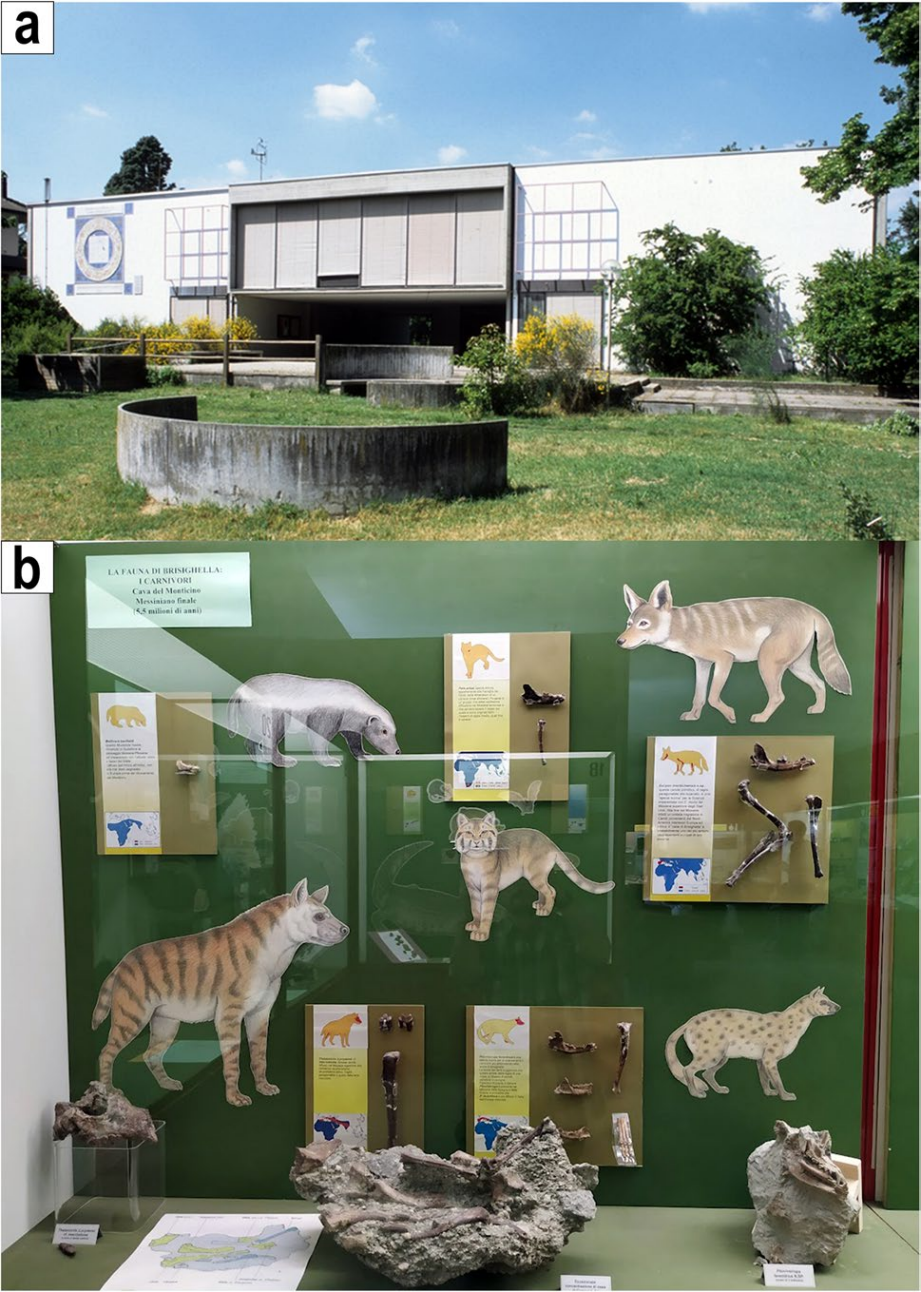
Civic Museum of Natural Science ‘D. Malmerendi’ of Faenza. **a** Outside view of the building from the botanic garden ‘Giardino Botanico Paolo Liverani’. **b** Example of the exhibits of the museum, particularly the carnivorans from the Late Miocene Cava Monticino site

The Natural Sciences Museum of the municipality of Faenza was founded in 1980 after the acquisition by the municipality of the zoological collections (specifically ornithological and entomological collections) of the surveyor Domenico Malmerendi (1900–1980). In the 1980s, thanks to collaboration with the local speleological group, the collections were enriched with geological and palaeontological specimens collected from the area surrounding Faenza and the whole Ravenna province. Together with fossils from the Pliocene marine deposits and the Quaternary alluvial successions, a large amount of rocks and fossils was collected from the widespread karstic cave system that characterises the area located in the north-western part of the ‘Appennino Tosco-Romagnolo’ (a portion of the Apennines between Tuscany and Emilia-Romagna—particularly the transregional border shared by the provinces of Florence and Ravenna) due to the extensive outcrops of the Messinian gypsums of ‘Gessoso-Solfifera Formation’ (deposited between 5.96 and 5.61 Ma during the Messinian Salinity Crisis; Marabini and Vai 1989). In this sector, this formation has the considerable height of 200 m, with extensive karst processes as interesting features. The fossils coming from this sector range in age from the latest Miocene (such as those from the Cava Monticino and its mainly clayish infilling of the ‘Colombacci Formation’; Marabini and Vai 1989; Sami 2021) to the late Pleistocene (Sami and Ghezzo 2015) (Fig. 2).

## Materials and Methods

### Selected Materials Digitised

From the extensive collections of the Geology and Paleontology Museum of Florence (IGF) and of the Natural Sciences Museum of Faenza (MSF), we selected a representative subset, in agreement with the curator of each museum, according to the museum and the scientific interest of the resulting 3D data obtained by the scans. In the case of IGF, the primary objective was to digitise the most scientifically and historically relevant specimens held in the collection. Therefore, the first fossils to be scanned included type specimens of renowned taxa (principally vertebrates) from the Miocene-Pleistocene of Italy (particularly from Tuscany). Among them are the famous types of the Upper Valdarno collections, which not only merge scientific significance but also are relevant historically: some of these very specimens are part of the history of Vertebrate Palaeontology depicted by the famous French palaeontologist G. Cuvier, who used them to erect new species in his work of the 1810s to 1820s. Another criterion that guided the selection was the historical importance of the specimen, such as those that were part of the bulk of the Medici’s private collection of fossils (thus known from at least the seventeenth century, if not earlier) (Monechi and Rook 2010) or the specimens used to erect taxa no longer considered valid. The third criterion used to select valuable specimens from IGF was to scan those with ‘iconic value’, such as sabertoothed cats and mammoths. These criteria met the requirements and availability of the museum staff in charge of supervision of the digitisation, which had the purpose of documenting, archiving and eventually sharing 3D models of the fossils with experts and the non-expert public.

In the case of MSF, the selected material consisted of the most relevant skeletal remains of the fauna from Cava Monticino (a latest Miocene karstic site located close to Faenza) and some of the ossiferous blocks found there. One of the main goals, apart from the creation of 3D copies of the specimens via surface scans, was to determine whether the ossiferous conglomerate included other bones and, if so, to non-invasively investigate them further with digital techniques.

### Digital Acquisition and Elaboration Methodologies

Digital models of the selected fossil specimens were obtained through non-invasive techniques of surface scanning and tomographic imaging. The former was carried out using three-dimensional (3D) high-resolution surface scanners that differed in their resolution, field of view, and portability, with the aim of obtaining the best results, depending on, for instance, the size of the specimen or the intended resolution of the final 3D files. Large- to medium-sized specimens were digitised using two hand-held scanners (the Artec Eva and Artec Space Spider), whereas the Artec Micro acquires detailed 3D models of small specimens. The specific parameters of each scanner reveal their scan capability of acquisition (https://www.artec3d.com).

The Artec Eva field of view (FOV) ranges between a maximum of 83.8 × 48.8 cm and a minimum of 24.4 × 14.2 cm with a working distance of 40–100 cm from the scanned object. This allows the Artec Eva to scan objects larger than 10–20 cm in total length. The maximum 3D resolution and mesh accuracy of the Artec Eva is 0.2 mm and its accuracy is 0.1 mm. Examples in which we used the Artec Eva were large specimens, such as those of *Mammuthus meridionalis* or *Hippopotamus antiquus* (IGF) and the bone breccias of Cava Monticino (MSF)*.* The Artec Space Spider provides higher resolution scans due to the closer proximity to the specimens (working distance 20–30 cm) and smaller FOV (9 × 7 cm and 18 × 14 cm). This close-range scanner is ideal for medium to small specimens (i.e. a maximum size of approximately 50 cm). Larger specimens can also be scanned, although the raw data created by these scans are often difficult to post-process using desktop or portable workstations (e.g. the size of the file, the number of required scans to cover the entire surface); thus, the use of the Artec Space Spider is not advised for large objects. The 3D resolution of the Artec Space Spider reaches 0.1 mm, whereas the 3D accuracy of the generated mesh is 0.05 mm. In our case studies, the Artec Space Spider was used for more detailed or smaller specimens, such as the cranial and dentognathic remains of *Megantereon cultridens*, *Canis etruscus* and *Oreopithecus bambolii* (IGF) or those of *Lycyaena* cf. *chaeretis*, *Eucyon monticinensis* or *Oioceros occidentalis* (MSF).

**Fig. 3:**
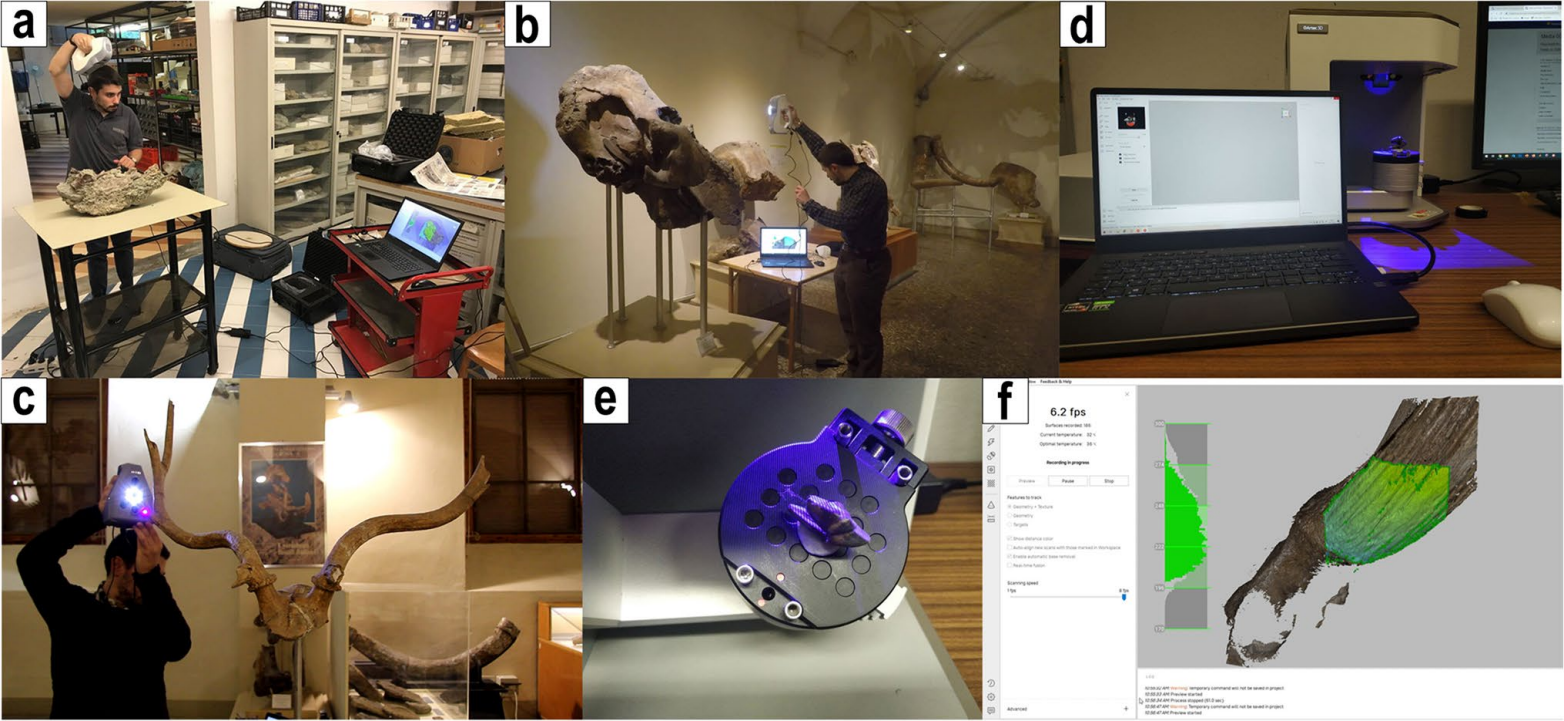
Scanning process with high-resolution surface scanners Artec Eva (**a**, **b**), Artec Space Spider (**c**) and Artec Micro (**d**, **e**). In **f**, the visualisation of the scans in the native software Artec Studio 15 Professional

The third scanner used in the digitisation process, especially for the material from Cava Monticino of the Museum of Faenza, was the desktop Artec Micro, which has the highest resolution and accuracy of all the scanners: 29 μm (the maximum 3D resolution) and 10 μm. Its specifics are therefore excellent for digitising very small objects (the maximum dimensions, according to the scanner specifics, are 6 × 6 × 9 cm). Indeed, we used the Artec Micro for type specimens of small mammals (e.g. those represented by isolated teeth), for tiny postcranial bones of birds and reptiles, and on specimens in which we were interested in obtaining a high-resolution mesh (e.g. peculiar enamel structures). All three scanners, both the hand-held (Artec Eva and Space Spider) and the desktop (Artec Micro) versions, use blue-light technology to map the surface geometry of the specimens, while capturing high-quality pictures of their texture with the specific cameras present on them. Nevertheless, the scanning procedure differs between the hand-held and the desktop scanners. For the Artec Eva and Space Spider, the selected object is scanned in multiple passes, taking care to acquire a significant portion of overlap between two scans (Fig. 3). This speeds up the postprocessing and 3D reconstruction process. With the Artec Micro, the scan acquisition is performed using the native software Artec Studio 15 Professional (ver. 15.0.3.425). The object is placed on an automated platform that rotates and tilts according to the selected scanning paths available in the program, thereby allowing the acquisition of the object’s surfaces from every desired angle. The paths can also be customised according to the researcher’s own needs (Fig. 3).

**Fig. 4:**
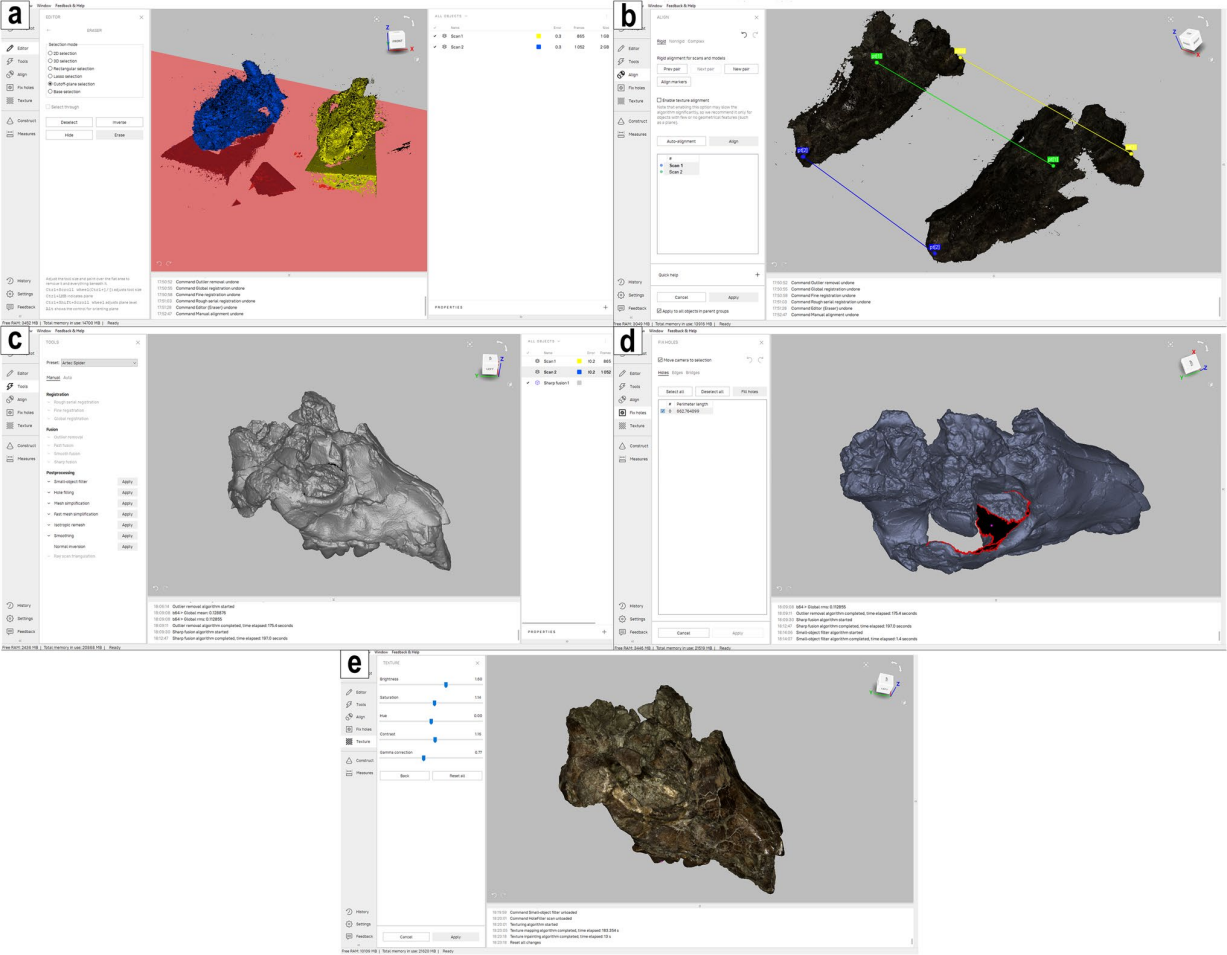
Workspace of the native software Artec Studio 15 Professional showing some of the most relevant steps from the acquired scan to the finalised mesh of a cranium of *Lycyaena* cf. *chaeretis*: **a** base removal, **b** alignment of the scans, **c** construction of the 3D mesh, **d** hole filling, **e** texture mapping on the obtained mesh

Regardless of the scanner used in the acquisition, the raw data are registered and processed using Artec Studio 15 Professional. The native software carries out all the steps required to obtain the digital surface reconstruction of the target specimens (Fig. 4), from editing of the single scans and their alignment and fusion to texture mapping on the 3D mesh and export of the final 3D model in the most common digital file formats (e.g..obj,.stl,.ply).

**Fig. 5:**
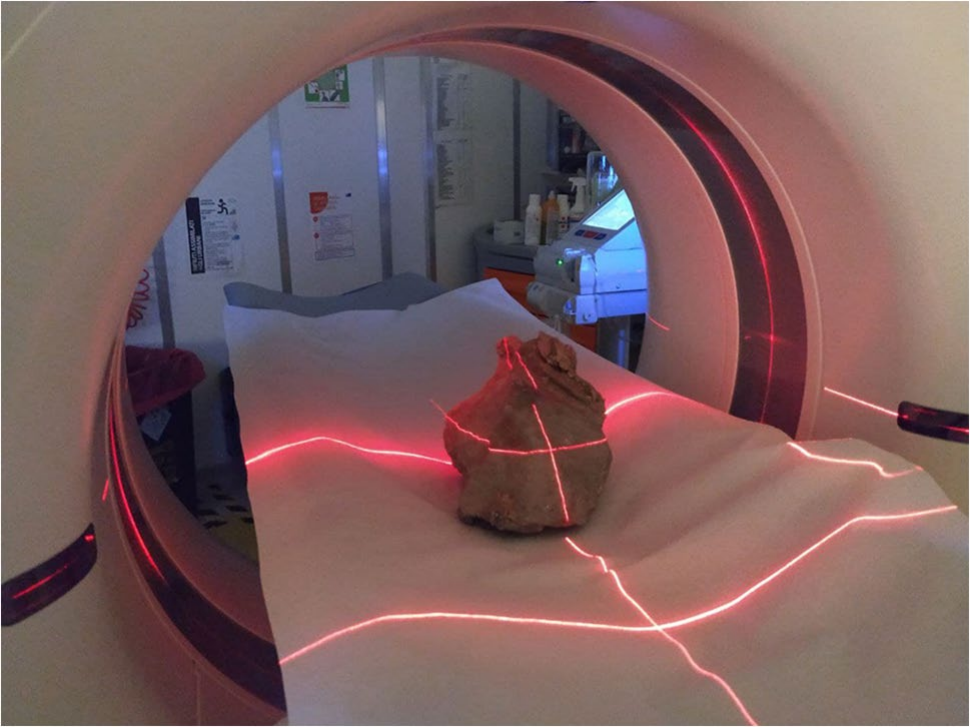
CT scan of the fossiliferous block MSF 62, which includes numerous bones of *Plioviverrops faventinus*, acquired at the Hospital San Giovanni di Dio of Florence

In the case of MSF, we were also able to apply tomographic imaging to two relevant ossiferous blocks (MSF 62 and MSF 89), which are particularly rich in bones, to investigate the presence of additional fossils contained within the sediment matrix and not visible on the surface of the block. The tomographic scans were performed at the Medical Radiology ward (‘SOS Radiologia’) of the San Giovanni di Dio Hospital (Florence) using a Siemens Somatom Definition AS scanner. To avoid excessive handling of the more fragile samples, those samples were scanned while inside the packaging used for their safe transport from MSF to Florence. The best-preserved samples were directly exposed to the scanner (Fig. 5). Different preset settings of the scanning program were used according to the size of the scanned samples to reach an optimal resolution for each sample.

The scan yielded 1392 images for ossiferous block MSF 89 and 758 for block MSF 62, each with a pixel size of 512 × 512, a thickness of 0.6 mm and a pitch of 0.4 mm. The output data were DICOM images (.dcm files). The samples were scanned twice with slightly different settings, particularly by changing the kernel parameter (i.e. the convolution matrix), to obtain two raw datasets of each piece with different blurring and sharpness. The data obtained from the scans were visualised with the free MicroDicom viewer software (ver. 3.9.5; https://www.microdicom.com/) while the processing of the DICOM files and the analysis were performed using Amira (ver. 5.4.5; https://www.thermofisher.com/order/catalog/product/AMIRA). The open-source Blender software (ver. 2.93; https://www.blender.org) was used to improve the visualisation of the 3D mesh resulting from the segmentation of the blocks.

### Augmented Reality Web App

Some of the 3D models resulting from the digitisation of the two collections were also implemented in augmented reality protocols to allow their remote visualisation simply using a mobile device (e.g. smartphone or tablet) with a camera and an internet connection. The selected specimens presented here include two of the most relevant fossils in the exhibitions of the IGF and MSF: (1) the almost complete skeleton of *Hippopotamus antiquus*, IGF 1043, the type specimens used by the French zoologist A. G. Desmarest (1784–1838) to erect the species; and (2) the small ossiferous block with the remains of several individuals of *Plioviverrops faventinus*, MSF 62. The created code-based web app follows the methodology applied to paleontological specimens by Bartolini-Lucenti et al. (2020, 2021), based on the source codes of Etienne (2017) and Carpignoli (2019). The coding was conducted using Visual Studio Code (ver. 1.41) implementing AR.js and A-Frame (ver. 0.9.2; https://www.aframe.io) to render the 3D model in the environment.

## Results and Discussion

**Fig. 6:**
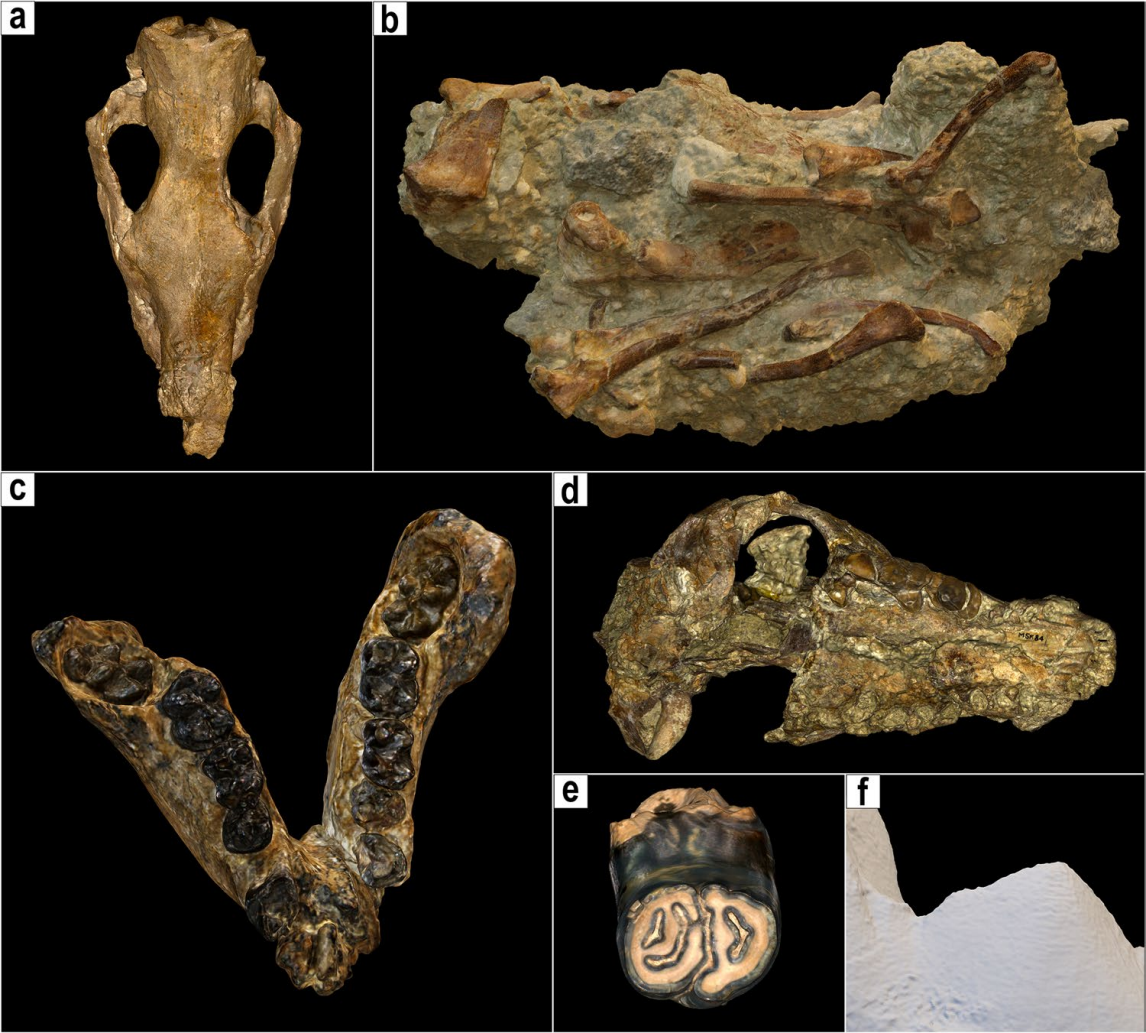
Results of digisation in both museums using different scanners. **a**, **b** Specimens scanned with Artec Eva (**a** *Stephanorhinus etruscus*, type cranium, IGF 756; **b** ossiferous block MSF 89); **c**, **d** specimens scanned with Artec Space Spider (**c** *Oreopithecus bambolii*, type juvenile mandibles, IGF 4335; **d** cranium of *Lycyaena* cf. *chaeretis*, MSF 84); **e**, **f** specimens scanned with Artec Micro (**e** *Hystrix etrusca*, incisor, IGF 938; **f** detail of the mesh of single m1 of *Eucyon monticinensis*, BRS 5/27). Specimens not to scale

In both case studies, the results of the digitisation were excellent in terms of the quality and fidelity of the final 3D meshes (Fig. 6), in terms of the satisfaction of the museum staff and in terms of the new opportunities opened for museum exhibitions and collection management.

**Fig. 7:**
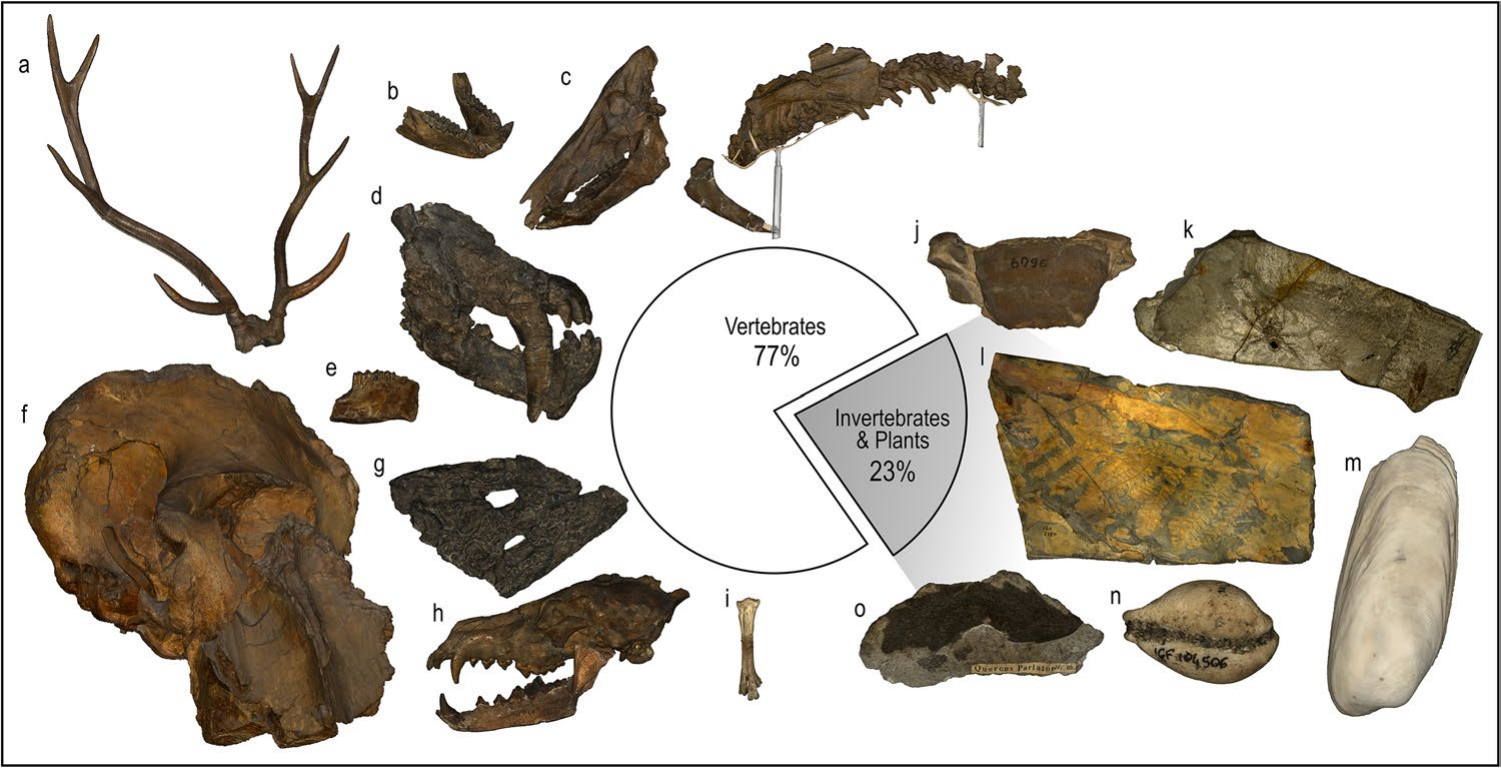
Proportion of scientifically and historically relevant specimens scanned at the Geology and Paleontology Museum of Florence. Selected examples: **a **cranium fragment with antlers of *Pseudodama nestii*; **b **mandibles of *Macaca sylvanus florentina*; **c** skull and partial skeleton of *Sus strozzii*; **d** broken skull of *Homotherium crenatidens*; **e **hemimandible fragment of *Lepus valdarnensis*; **f **almost complete cranium of *Mammuthus meridionalis*; **g **fragment of cranium of *Crocodylus bambolii*; **h **skull of *Canis arnensis*; **i **tarsometatarsus of *Fuligula aretina*; **j **carapace of *Gonoplax meneghiniii*; **k **slab with *Argyroneta destefanii*; **l **slab with several fronds of *Acitheca isomorpha*; **m** shell of *Coralliophaga brocchii*; **n** shell of *Cypraea haveri*; **o** leaf of *Quercus parlatorii*

The scanning activity at IGF has resulted in the digitisation of a total of nearly 300 specimens. Most of the selected samples (more than 75%) are vertebrates, whereas the remainder consists of invertebrates and plants (Fig. 7). The vertebrate fossils include type and historically relevant specimens of mammals (skeletal and dental material), reptiles (skeletal, dental and ichnological material), birds (skeletal material) and chondrichthyans (dental material). They span a stratigraphic range from the Middle-Late Triassic (with the amniotes’ footprints of Monte Pisani) to the Upper Pleistocene, although most of the specimens are Late Neogene-Quaternary in age (Monechi and Rook 2010). Regarding the invertebrates, the majority are mollusc shells (mainly Cenozoic bivalves and Mesozoic cephalopods), followed by corals, plants, arthropods, echinoderms and brachiopods. The scanned types of plants come from the relatively abundant fossil flora of the Carboniferous-Permian deposits of Monti Pisani (Monechi and Rook 2010). The 3D models constitute the results of this first digitisation of a nationally and internationally relevant palaeontological collection in Italy. They will compose the bulk of the digital archive, and the museum staff is currently working on a new online platform to host, visualise and share the models with researchers interested in studying them, as well as with the widest possible public audience, encompassing anyone fascinated by palaeontology and evolution.

**Fig. 8:**
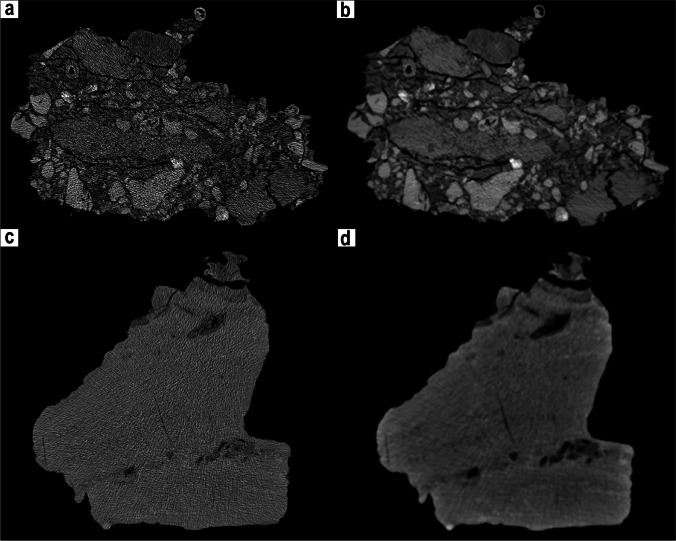
Raw data of the CT scans of MSF 89 (**a**, **b**) and MSF 62 (**c**, **d**) using different kernel values. The sets in **a** and **c** have higher values, so sharper scans, whereas **b** and **d** have lower kernel values, so more blurred. The use of these different presets in the scans deeply affects the possibility of discriminating between bone, matrix, and clasts

The second case study involved the use of both surface and CT scans of the material from the Miocene site of Monticino gypsum quarry at Brisighella (Faenza, RA, Italy) held at MSF, with a particular focus on two exhibited fossiliferous blocks (MSF 62 and MSF 89). These specimens have slightly different compositions: one is a compact clayish-sandy block with the bones of a small Miocene hyaenid (*Plioviverrops faventinus*) and two others are argillitic-chalky microconglomerates that contain numerous bones of carnivorans and herbivores of the diverse association of Cava Monticino within them (Rook 2021) (Fig. 8). Apart from the bones emerging on the surface of the conglomerates, the possibility of finding and identifying bones hidden within the blocks has never been investigated by researchers or by the museum staff. Furthermore, the peculiar composition of the conglomerates makes the blocks particularly fragile so they could easily deteriorate. The digital applications allow the preservation of the specimens in their existing state by surface scanning and determining the presence of bones inside the blocks using tomographic imaging. Indeed, both specimens contained bones embedded in the matrix. Despite this interesting result, the digital extraction of bones from the matrix or from the other clasts surrounding the bones of MSF 62 and MSF 89 was particularly complex and yielded different results for the two blocks. The density of the fossilised bone appeared to be incredibly similar to that of the sediment (as in the case of MSF 62) and to the clasts that make up the microconglomerate of block MSF 89 (Fig. 8). The quality of the slices did not improve, even when applying different kernels (Fig. 8). For high values of the convolution matrices (Fig. 8), the sharpness correction applied by the scanning program generated ‘interferences’, which disturbed both the visualisation and the elaboration. Similar problems arose with low kernel values (Fig. 8), as the contours of the various bones appeared too smoothed and blurred, so they were difficult to make out, as they were less defined and marked than the surrounding sediment. These undesired effects of the pattern of interference and blurriness of some components were particularly strong for MSF 89, where the sedimentological-chemical composition of the microconglomerates and the large number of clasts made segmentation of the acquired data virtually impossible.

**Fig. 9:**
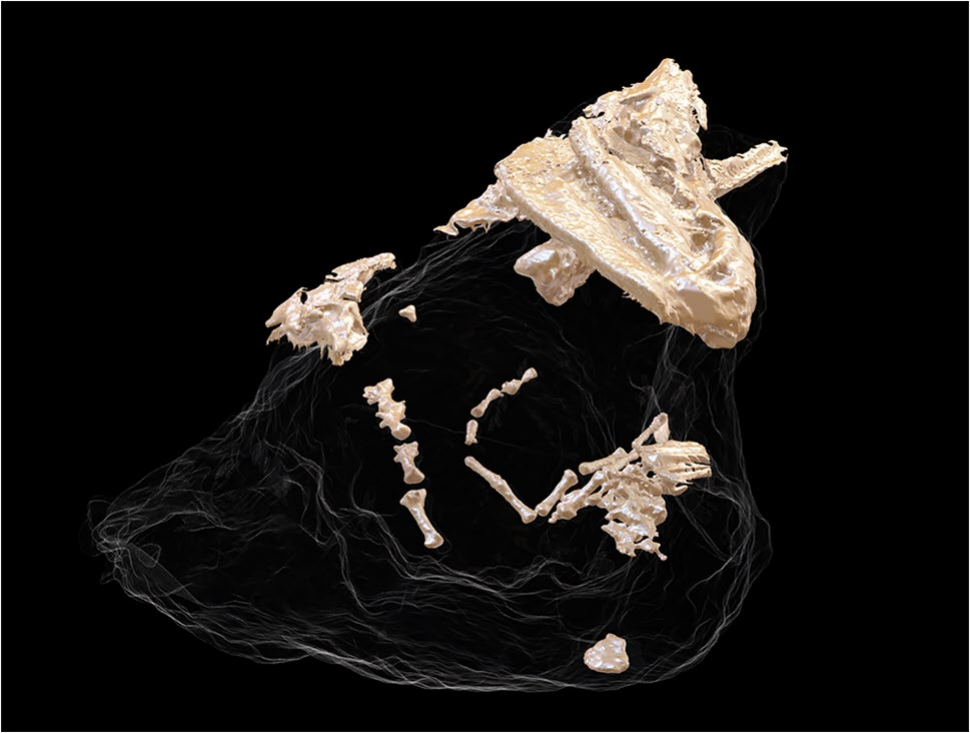
Image obtained thanks to the segmentation of CT data of the sandy-clayish block MSF 62 showing the fossil bones preserved within the block itself

Despite these difficulties, at least in the case of MSF 62 (the smaller specimen with the cranium of *Plioviverrops*), we were able to obtain 3D models of the previously unsuspected numerous bones contained within the block itself (Fig. 9). Most of these consist of postcranial bones, mainly caudal vertebrae and metapodia, of a small carnivore, presumably *Plioviverrops*, and of a medium-large carnivore, possibly the hyaenid *Lycyaena* cf. *chaeretis* (as no larger carnivorans are currently known from Cava Monticino; Bartolini-Lucenti et al. 2022). Further detailed studies of these materials are currently underway. Regardless, the tomographic investigation has allowed us to show, for the first time and while retaining the total safety of the samples, that the fossil content of these blocks is more abundant and even more interesting than expected from an external examination. These results were presented at the Museum of Faenza in the second half of 2021 at two different public meetings, which were attended by a large audience. The curator of the museum, surprised by the results of the non-invasive techniques, is planning to include digital screens and 3D prints in the current exhibition to inform visitors of the even greater value of these beautiful specimens preserved and displayed in the museum.

### The use and future possibilities of the digital fossils in the case studies

**Fig. 10:**
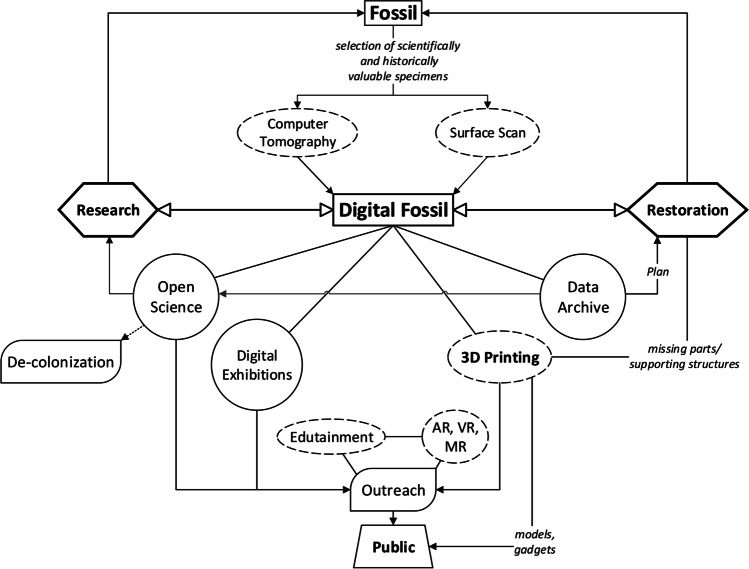
Virtual paleontology workflow followed in the two digitisation projects here presented, with the advantages prospected by the resulting 3D models of fossils. Legend: rectangles = (physical or digital) objects; dashed ovals = techniques and methods (3D printing is reported in bold to represent its relevance as an advantage offered by digital fossils but also the outstanding variety of methodologies, types, material nowadays available as 3D prints); hexagons = different branches of paleontology; circles = opportunities; rounded parallelograms = practices (e.g. decolonisation, repatriation) or topics relevant to museology (e.g. outreach). Lines and arrows indicate (some of) the possible connection between the topics as deepened in the text

Despite the differences between the two museums and the specific objectives of these applications, many benefits in common are evident resulting from the use of 3D scans. Other authors have underlined these advantages in general terms (e.g. Cunningham et al. 2014; Adams et al. 2015), but we will discuss them here with regard to the two considered contexts. Figure 10 sums up both the workflow and the aforementioned opportunities. In conservation, reports of the state of preservation of a specimen are essential to understand whether and how to handle it, to know its stability and, eventually, to plan its restoration. Written notes and photographs remain valid and irreplaceable means of registering this information. However, with digital techniques, the amount of data recorded in a single session of scans (especially tomographic ones) is far larger and more complete than any ‘analogue’ means of data collection. In terms of planning, 3D copies of the fossil specimens offer the chance to see the results of the restoration directly on the digital object simply by modelling it into the restored version. In this way, curators and technicians could approve or disregard interventions that would otherwise alter the original specimens. Today, numerous digital methodologies are available that could be used to retrodeform taphonomically altered fossils (see Cirilli et al. 2020). Together with the museum staff, we created a database of 3D models and raw data to store the digital information of each specimen, recording the current state of preservation of invaluable fossils like the type specimens of IGF or the large ossiferous blocks of MSF. Especially in the latter cases (and similarly, whenever specimens are considerably fragile), their superficial and tomographic data, as well as their 3D reconstruction, could prevent the loss of their original structure.

At the same time, these data allow us to consider restoration intervention and/or extraction of the bones embedded in the sediment (like those shown in Fig. 9). A relevant alternative to the physical extraction of the bones enclosed within the block would be to print them, thereby allowing researchers and visitors at the MSF to touch and manipulate them, while keeping the fragile specimens safely in their showcases. This is an interesting and commonly appreciated way of expanding the standard exposition (as evident by surveys such as that of Wilson et al. 2017 and references therein) to enable a more interactive and intuitive experience of the museum, with attention to visitors with vision disabilities who could then also enjoy the museum collection.

The construction of the database of digital data is also relevant, as the museum staff at IGF is also planning the use of online platforms, owned and controlled by the museum or via existing clients, to host and make the models available on request to scientists, teachers or students interested in them. Indeed, one of the key advantages of using digital fossils is the opportunity for good practices of open science. As with any other type of digital file, 3D models of fossils can be exchanged as easily as sending an email. This has great potential for the entire palaeontological community, allowing researchers from distant places to visualise and study fossil specimens without the cost of travel and accommodation to reach the collections.

With virtual palaeontology becoming a common practice in palaeontological analyses, the issue of copyright and/or ownership of the perfect copies of the physical specimen has arisen (Lewis 2019). Museum institutions that already use online repositories (e.g. Digimorph, Morphosource) to store and share their digital models now use ad hoc one-to-one agreements via online/digital forms, which need to be filled in and submitted to the curators before authorisation for the download is granted (see, e.g., Adams et al. 2015). The curators of IGF, together with our help and that of the legal office of the University of Florence, prepared a similar document for incoming visiting researchers who wish to make use of 3D acquisition methodologies and for those who require 3D models of some fossils. The great advantage is the use of the models in virtual exhibitions, both in situ and online. Many scientific museums today use websites or mobile apps that allow remote visits to their exhibitions and often implement 3D models.

The curators of IGF are currently developing an online experience of a 360° virtual visit to the museum, which will include the digitised models resulting from the project presented here. This online exhibition should be released sometime during 2023. Digital models could also be effectively used on site as parts of virtual reality (VR), augmented reality (AR) and mixed reality (MR) exhibits. Of the three, AR experiences are generally intuitively easy-to-use and attractive to user of all ages, more and more often available for ludic or educational purposes (Cabero-Almenara et al. 2019), as testified by the increasing number of AR apps on the online store repositories (i.e. App Store and Google Play Store; Koetsier 2017). Moreover, AR gives the visitor the liberty to experience the exposed material and the digital implementations at their own pace, without the use of immersive or special devices other than one’s own tablet or smartphone (Azuma 1997; Rigby and Smith 2013; Yuen et al. 2013; Akçayır and Akçayır 2017; Fistola et al. 2020). It has the asset without replacing neither the physical world nor the real object with digital copies, as VR (Bennettet al. 2022), but rather expanding the potentiality of the exhibitions with additional resources. Several authors have discussed the benefit effects that AR have to count the ‘digital isolation’ involving the user in first person (despite the actual objective of the AR experience, e.g. educational, edutainment, game) (Pyae and Potter 2016; Cabero-Almenara et al. 2019 and reference therein). Of the many types of AR methodologies (e.g. Bower et al. 2014; Kolivand et al. 2018), marker-based application has proven suitable for independent and remote use by users (Kan et al. 2011; Cabero-Almenara et al. 2019). For instance, in the case of the results of scientific research (as in Bartolini-Lucenti et al. 2020, 2021), the AR content can be used to perform intuitive digital comparisons between different specimens. In a similar way, scientific museums could take advantage of the high appeal of digital fossils to boost their visibility and engagement with the general public, enabling people to visit exhibitions on the web or for visitors to experience the museum collections in an innovative way (e.g. Duguleana and Voinea 2018; Bennett et al. 2022).

**Fig. 11:**
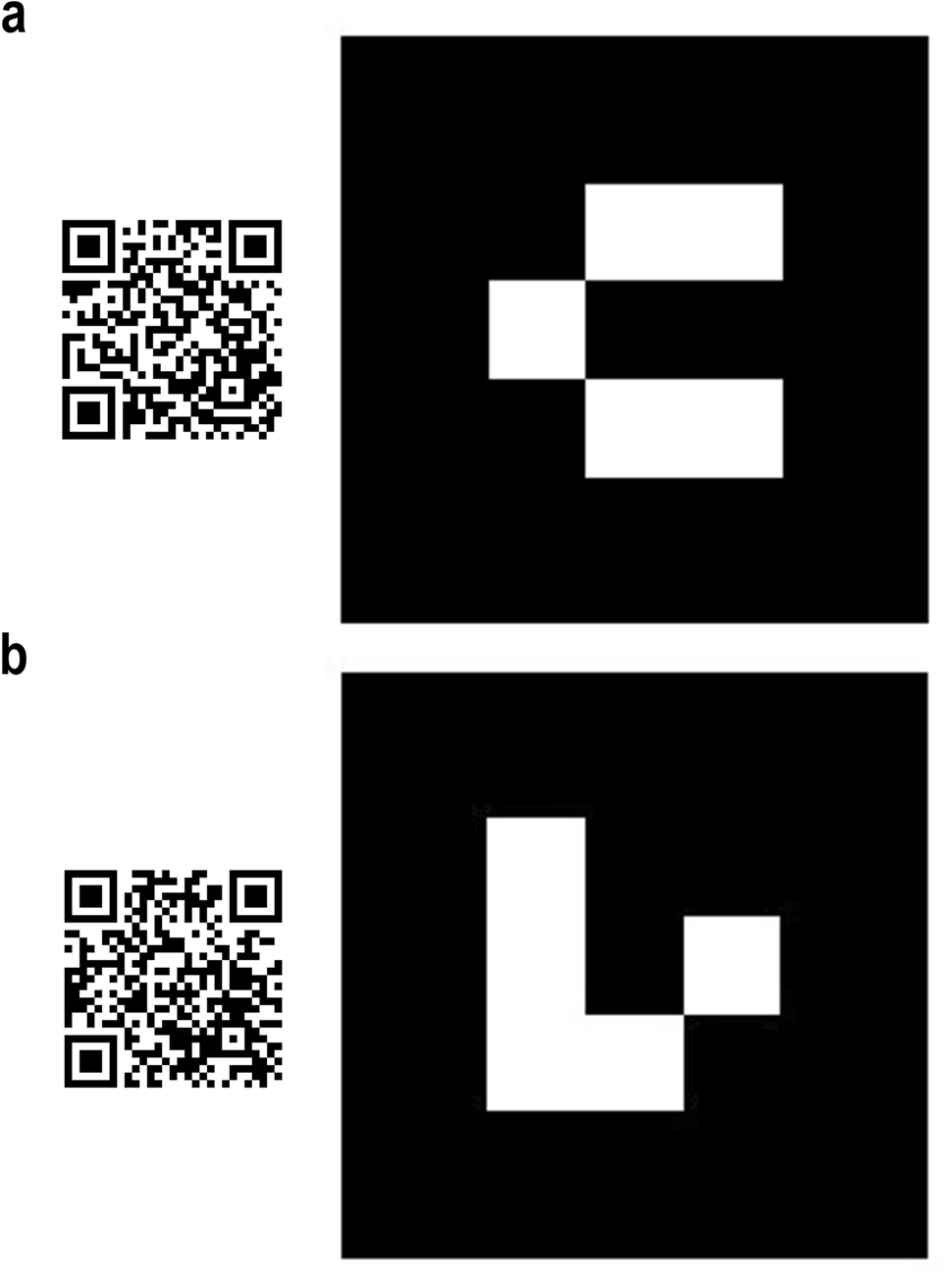
QR code and augmented reality (AR) marker showing two models: **a** the *Hippopotamus antiquus* skeleton (IGF 1043) of the Geology and Paleontology Museum of Florence and **b** the clay block with *Plioviverrops faventinus* (MSF 62) from the Civic Museum of Faenza. Instructions: Scan the QR code on the left; open the link; allow the browser to access the camera of your device; point the camera toward the marker (on the right); and wait for the model to load (up to 10 s). It is possible to turn the device around the marker (or to move the marker) to see different parts of the model. Best visualisation performances can be achieved by printing the markers, rather pointing at them on screens. Refer to Appendix and Bartolini-Lucenti et al. 2020; 2021) for common issues

In our projects, a marker-based AR application was also employed in the cases of IGF and MSF to engage the public by showing the ossiferous block MSF 62 and the mounted skeleton of *Hippopotamus antiquus* during the COVID-19 pandemic. Figure 11 shows the two web apps and AR renderings of these 3D models, using a QR code (to access the webpage of the AR content) and a marker. On site, an easy-to-use marker-based or georeferenced AR web app could be implemented in the exhibition itinerary to show hidden contents (e.g. the embedded bones of MSF 62 or the internal volumes and structures of fossils of IGF), thereby increasing the attractiveness of the museum with limited cost or resource expenditure.

## Concluding Remarks

The use of virtual palaeontology methods in museums is a valuable and progressively more affordable possibility that enhances the engagement and awareness of non-expert visitors, while also favouring conservation and open-science virtual practices. Moreover, systematic digitisation projects, such as the ones we carried out at the Geology and Paleontology Museum of Florence and the Natural Science Museum of Faenza, follow the guidelines and objectives of the 2019 European Commission Museum ‘*Declaration of Cooperation on advancing the digitisation of cultural heritage*’ to promote the digitisation of European cultural heritage, thereby respecting the goals of many museal institutions. Sparked by the scanning activity in their collections, as preliminarily reported here, both museums are starting to plan and create virtual environments and applications, such as the creation of an online platform hosting the 3D models and a new virtual tour of the exhibition of the Geology and Paleontology Museum of Florence, as well as the possible implementation of 3D prints and/or screen showings of digital fossils to the public in the Natural Science Museum of Faenza.

## Appendix: Guide to use of the AR web app

To visualize the QR code: choose any free application from the App Store/Play Store. Best performance of the AR visualisation can be achieved with printed markers, rather than on screens (pc monitors, tablets, etc.). Prints reducing back-scattered light on the markers represent optimal conditions for the AR visualisation.

## iOS devices

To visualize the Augmented Reality web app:

1. Allow Safari to open the camera of the iPhone: Go to Setting > Safari > turn on ‘Camera and Microphone Access’ under the ‘Privacy and Security’ submenu.
2. Scan the QR code.
3. Open the link in Safari (n.b.: the web app does not work in Chrome for iOS or other browsers).
4. Confirm the use of the camera by the browser.
5. Point at the marker, wait for the model to load.

Best model-rendering performances if the scene is in landscape mode (i.e. allowing the phone to automatically rotate the view). N.b. If the model does not appear in landscape mode, although it was visible in portrait mode, refresh the page. Recommended iOS versions to visualize the AR content 15.0 or above.

## Android devices

To visualize the QR code: choose any free application from the App Store.

To visualize the Augmented Reality web app:

1. Scan the QR code.
2. Open the link in your mobile browser (note that Chrome is preferable).
3. Confirm the use of the camera by the browser.
4. Point at the marker, wait for the model to load.

Best model-rendering performances if the scene is in landscape mode (i.e. allowing the phone to automatically rotate the view). N.b. If the model does not appear in landscape mode, although it was visible in portrait mode, refresh the page. Additionally, some users may need to install ‘Google VR services’ to their mobile phones in order to see the AR content.
